# Sex differences in substance use, health, and social functioning among opioid users receiving methadone treatment: a multicenter cohort study

**DOI:** 10.1186/s13293-015-0038-6

**Published:** 2015-11-10

**Authors:** Monica Bawor, Brittany B. Dennis, Michael Varenbut, Jeff Daiter, David C. Marsh, Carolyn Plater, Andrew Worster, Meir Steiner, Rebecca Anglin, Guillaume Pare, Dipika Desai, Lehana Thabane, Zainab Samaan

**Affiliations:** MiNDS Neuroscience Graduate Program, McMaster University, Hamilton, Ontario Canada; St. George’s, University of London, London, UK; Population Genomics Program, Chanchlani Research Centre, McMaster University, Hamilton, Ontario Canada; Peter Boris Centre for Addiction Research, St. Joseph’s Healthcare Hamilton, Hamilton, Ontario Canada; Department of Clinical Epidemiology and Biostatistics, McMaster University, Hamilton, Ontario Canada; Canadian Addiction Treatment Centres (CATC), Richmond Hill, Ontario Canada; Department of Medicine, McMaster University, Hamilton, Ontario Canada; Northern Ontario School of Medicine, Laurentian Campus, Sudbury, Ontario Canada; Department of Psychiatry and Behavioural Neurosciences, McMaster University, Hamilton, Ontario Canada; Women’s Health Concerns Clinic, St. Joseph’s Healthcare Hamilton, Hamilton, Ontario Canada; Department of Obstetrics and Gynecology, McMaster University, Hamilton, Ontario Canada; Biostatistics Unit, Centre for Evaluation of Medicine, Hamilton, Ontario Canada; Mood Disorders Program, St. Joseph’s Healthcare, 100 West 5th Street, Hamilton, Ontario L8N 3K7 Canada

**Keywords:** Substance use disorders, Opioid addiction, Methadone maintenance treatment, Sex differences, Women’s health

## Abstract

**Background:**

Despite the growing numbers of men and women with opioid use disorder in Canada, sex-specific issues in treatment have not been re-examined in the current population of patients with opioid addiction. We aimed to evaluate sex differences in substance use, health, and social functioning among men and women currently receiving methadone treatment for opioid use disorder in Ontario, Canada.

**Methods:**

We recruited 503 participants with opioid dependence disorder receiving methadone maintenance treatment. We collected data on demographics, treatment characteristics, psychiatric history, addiction severity, and drug use patterns through urinalysis. We performed adjusted univariate analyses and logistic regression to identify distinct factors affecting men and women.

**Results:**

Among our sample of 54 % (*n* = 266) men and 46 % women (*n* = 226) with mean age 38.3 years, less than half of participants were employed (35.6 %) and married (31.8 %) and had completed a high school education (27.9 %). Compared to men, women had frequent physical and psychological health problems, family history of psychiatric illness, and childcare responsibilities and began using opioids through a physician prescription. Men had higher rates of employment, cigarette smoking, and cannabis use compared to women.

**Conclusions:**

Our results have revealed different patterns of substance use, health, and social functioning among men and women currently receiving methadone treatment for opioid addiction in Ontario, Canada. This information can be used to develop an integrative treatment regimen that caters to the individual needs of men and women, as well as to inform methadone treatment protocols to include specialized services (including vocational counseling, childcare and parenting assistance, medical assistance, relationship or domestic violence counseling, etc.) and increase their availability and accessibility on a larger scale.

## Background

The last decade has witnessed significant changes in patterns of illicit opioid use in Canada [1]. Increases in the availability and utilization of opioids for the management of pain conditions in primary care settings [2] have resulted in the shift from heroin use to non-medical prescription opioid use [3]. The number of opioid prescriptions has more than doubled over the last two decades [4, 5] and has been associated with a significant burden of opioid-related mortality nationwide with highest rates reported in Ontario [6–8]. Currently ranking first in global opioid analgesic consumption [9], Canadians are at a heightened risk for opioid abuse and dependence, giving rise to a major public health crisis [10].

Higher rates of prescription opioid use among women have been consistently documented across studies in Canada and the USA [3, 11–14]. Patterns of opioid prescribing are higher among women [15], who are more likely than men to suffer from poor health including pain conditions [16], making them especially vulnerable to misuse prescription narcotics. Indeed, the number of women seeking treatment for opioid-related disorders has markedly increased since the 1960s, reaching current levels that are comparable to men [17].

With the rising number of women seeking treatment for opioid-related problems, there is growing need for a re-evaluation of sex and gender differences in opioid dependence and treatment. Methadone maintenance treatment (MMT) is the most common form of opioid agonist therapy implicated for the management of opioid use disorders and currently serves over 35,000 patients in Ontario alone, a pronounced increase compared to 7800 in 2001 [18–20]. However, most of what we currently know about methadone treatment is based primarily on studies that included few or no women at all [21, 22]. Existing treatment options remain targeted towards opioid users of the past; primarily young, inner-city, heroin-injecting men. Despite the demographic transformation of this population, available prevention strategies and treatment options have not been revised to accommodate these developments.

The identification of these sex- and gender-specific patterns has been imperative for informing standards of care and clinical practice thus far, however many of these studies were completed in the 1990s and are not representative of today’s population of opioid users, nor have they accounted for advancements in assessment tools and research methodology. There is a critical need for a thorough re-evaluation of sex-related factors for men and women with opioid use disorder. Our objectives in this study are to (1) provide an updated and extensive description of the current population of opioid users in methadone treatment in Ontario, Canada, and (2) evaluate sex differences in substance use, health status, and social functioning among men and women currently receiving methadone treatment for opioid use disorder.

## Methods

### Study design and participant recruitment

We collected data for this study as part of the Genetics of Opioid Addiction (GENOA) research program, in collaboration with Canadian Addiction Treatment Centres (CATC; formerly known as Ontario Addiction Treatment Centres or OATC) and the Population Genomics Program (PGP) at McMaster University. Details of study methods have been reported previously [23–25]. We have since expanded our recruitment setting to include 13 opioid agonist treatment clinics.

We screened all eligible candidates for study inclusion. Participants were included in the study if they were ≥18 years of age, meeting criteria for DSM-IV opioid dependence disorder, attending CATC clinics for methadone treatment, and able to provide written consent and blood samples. Participants attending the clinics for opioid agonist treatment other than methadone were not eligible for this study. We chose to include only patients who are receiving methadone treatment as this is the most common opioid agonist treatment in Canada and is covered by most provincial health insurance plans, which allows us to recruit the largest sample possible. Other opioid maintenance treatments (e.g., buprenorphine, naltrexone) are less commonly used and also have distinct biochemical and physiological properties, which would increase the heterogeneity among the sample and render our findings less applicable to the opioid patient population as a whole.

Upon agreeing to participate in the study, participants provided informed consent and underwent baseline assessment, which consisted of a structured clinical interview administered by trained research staff. We collected self-reported data on demographics, treatment characteristics, age of initial opioid use, and psychiatric history. We also collected information on drug use patterns, measured through urinalysis, and addiction severity across multiple domains using the MAP tool. This study was approved by the Hamilton Integrated Research Ethics Board (HIREB; Study ID 11–056).

### Maudsley Addiction Profile (MAP)

We used the MAP instrument [26] to measure functioning across several life domains related to addiction; substance use, physical and psychological health symptoms, health risk behavior, and social functioning. The MAP evaluates numerous outcomes, which are common indicators of treatment performance in substance use disorders. Outcomes are evaluated based on the previous month. Originally developed in 1998 for patients with substance use disorders in the UK, it is now widely used and has demonstrated internal reliability and validity [26].

Data on substance use (including alcohol, heroin, illicit methadone, illicit benzodiazepines, cocaine/crack, amphetamines, and cannabis) including the number of days of use, amount, and route of administration was collected for the previous 30-day period. The health risk behavior domain assessed injection drug use, including number of days and frequency of sharing injecting equipment, as well as sexual behavior, including frequency of unprotected sex and number of sexual partners in the previous 30-day period. Frequency of physical and psychological health symptoms were assessed on a scale ranging from “Never” to “Always”; these responses were tabulated into a single score out of total score of 40, with higher values indicating more frequent health problems. The social functioning domain consisted of interpersonal conflict (days of contact and conflict with partner, family, and friends; represented as a proportion of days of conflict over days of contact in the analysis), employment (days employed and days missed from work), and criminal activity (number of days committed crime and number of times daily). Crime included selling drugs, fraud/forgery, shoplifting, and theft from property or vehicle. For analysis purposes, these were combined into a single variable representing *any* crime.

### Substance use

We collected data on weekly/bi-weekly qualitative and semiquantitative urine analysis using the iMDx™ Analyzer and Prep Assay (NOVX Systems Inc, Richmond Hill, Ontario, Canada). Urine drug screens are used as part of the clinical care model to monitor methadone adherence, as well as to identify the use of illicit opioids and other substances of abuse (including cocaine, cannabis, and benzodiazepines). The cut-off concentrations for detection by urinalysis were the following: 300 ng/ml for opiates, benzodiazepines, benzoylecgonine (cocaine metabolite), 100 ng/ml for oxycodone, and 50 ng/ml for tetrahydrocannabinol (THC). The iMDx™ assay is designed to distinguish between opioid classifications, including naturally-occurring and synthetic opioids. Urine samples were obtained and analyzed by trained clinic staff at the methadone clinic sites.

In this study, substance use behavior (including opioids, amphetamines, benzodiazepines, cannabis, and cocaine) was measured as the percent of positive urine screens per total number of available urine screens for each respective drug of interest over the previous three-month period. Alcohol abuse and dependence were measured according to the Mini International Neuropsychiatric Interview (M.I.N.I.) Version 6.0 [27]. Self-reported drug use in the past 30 days was collected using the MAP.

### Statistical analysis

We summarized descriptive sample characteristics using mean (standard deviation, SD) for continuous measures and number (percentage) for categorical variables. For variables with non-normal distributions, we reported median and interquartile range (*Q*_1_ and *Q*_3_). We performed adjusted univariate analyses on substance use behavior, health symptoms, and social functioning to test differences between men and women (defined as their biological sex) using multivariable linear regression for continuous variables and logistic regression for binary variables, while controlling for age, methadone dose, and duration of methadone treatment. Variables with non-normal distributions were log transformed prior to the analysis and differences were reported on the log scale. The primary outcome was opioid use measured through urine drug screening; all other outcomes were secondary. We used the false discovery rate (FDR) [28] method to control type 1 error rate when performing multiple comparison and adjusted *p* values accordingly. A sensitivity analysis was completed using the self-reported MAP assessment to measure substance use compared to urine drug screening. Regression model estimates including odds ratio (OR) for binary variables, mean difference (MD) for continuous variables, 95 % confidence intervals (CI), and *p* values (adjusted for covariates and multiple testing) are reported.

We did not employ imputations for missing data in our analysis as the proportion of missing data was negligible (4.1 %) [29]. Our sample size was adequately powered to perform multivariable logistic regression with 10 events per variable and 16 covariates in a sample of 226 women [30]. We used STATA Version 12 (StataCorp LP, College Station, USA) for all statistical analyses and we reported this study in adherence to the Strengthening the Reporting of Observational Studies in Epidemiology (STROBE) guidelines [31].

## Results

**Fig. 1:**
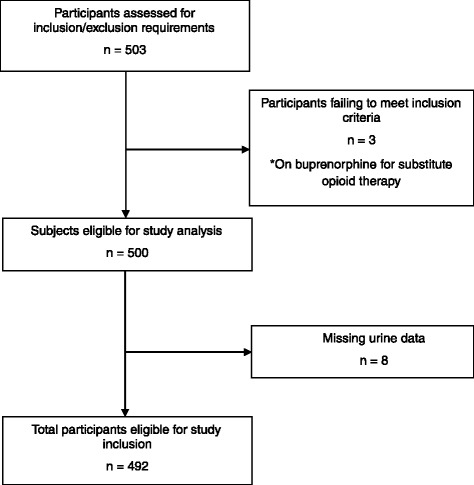
Eligibility and screening of candidates for inclusion in the GENOA study

We recruited a total of 503 participants receiving opioid agonist treatment from 13 CATC clinics. Among them, three participants were excluded because they had switched to treatment with buprenorphine rather than methadone. Further, eight participants were excluded as a result of failure to obtain blood and urine samples. A total of 492 participants were included in subsequent analyses (Fig. 1).

### Demographic and clinical characteristics

Our sample consisted of 54 % (*n* = 266) men and 46 % women (*n* = 226), with a mean age of 38.3 years and mean methadone dose of 77.6 mg (SD = 44.1). Less than half of participants were employed (35.6 %) and had completed a high school education (27.9 %). Age of initial regular opioid use was 25 years and age of first entry into methadone treatment was 32.2 years among the total sample. Almost half (44.2 %) reported that their first contact with opioids was through a doctor’s prescription for a medical illness (Table 1).

**Table 1:** Demographic and clinical characteristics of opioid-dependent men and women receiving methadone treatment

		Sex	
Characteristic	Total	Men	Women
	*n* = 492	*n* = 266	*n* = 226
Age, years; mean (SD)	38.3 (11.0)	39.5 (11.7)	36.9 (9.9)
European ethnicity; *n* (%)	393 (80.9)	219 (83.0)	174 (78.4)
Completed high school education; *n* (%)	136 (27.9)	65 (24.6)	71 (32.0)
Substance use and treatment history			
Age of initial opioid use, years; mean (SD)	25.0 (8.7)	24.9 (9.2)	25.1 (8.0)
Physician prescribed first opioid use; *n* (%)	217 (44.2)	100 (37.7)	116 (51.6)
Daily methadone dose, milligram; mean (SD)	77.6 (44.1)	81.3 (48.3)	73.3 (38.3)
Age of first MMT, years; mean (SD)	32.2 (9.6)	32.7 (10.1)	31.5 (9.0)
Duration of MMT, months; mean (SD)	51.6 (49.3)	52.9 (50.7)	49.9 (47.7)
Previous treatment, any; *n* (%)	149 (30.7)	90 (34.2)	59 (26.6)
Previous MMT treatments, number; mean (SD)	1.5 (1.1)	1.6 (1.1)	1.4 (0.9)

*SD* standard deviation, *MMT* methadone maintenance treatment

Women were younger than men (36.9 years vs. 39.5 years) and receiving a lower methadone dose (73.3 mg vs. 81.3 mg). Women also more commonly reported having had their first contact with opioids through a physician prescription (Table 1). Men and women were similar in their age of first regular opioid use, duration of treatment, and number of previous treatments for opioid use disorder.

### Substance use behavior

We collected data on participants’ substance use using urine toxicology screens and self-reported assessment with the MAP. Apart from cigarette smoking, which was prevalent in the majority of our sample (84.1 %), cannabis and alcohol were the most commonly reported substances of use within the past month (47 and 46 %, respectively), followed by cocaine (18 %) according to the MAP (Table 2). Alcohol abuse and dependence were diagnosed using the M.I.N.I. in 9.5 % of the entire sample. In the previous three months, the percentage of participants with substance use measured by urine toxicology was highest for opioids (48.5 %), followed by benzodiazepines (39.6 %), cocaine (34.7 %), and cannabis (23.1 %) (Table 2).

**Table 2:** Substance use behavior among men and women

		Sex		Adjusted analyses, men vs. women		
Outcome	Total	Men	Women	OR/MD	95 % CI	Adjusted *p*
	*n* = 492	*n* = 266	*n* = 226			
Primary						
Opioid use in prior 3 months, urine screening; *n* (%)	239 (48.5)	129 (48.5)	110 (48.5)	1.03	0.71, 1.50	0.911
Secondary						
Proportion of use in prior 3 months, urine screening; *n* (%)						
Amphetamines	23 (4.7)	15 (5.6)	8 (3.5)	0.68	0.28, 1.66	0.616
Benzodiazepines	195 (39.6)	95 (35.7)	100 (44.1)	1.60	1.10, 2.33	0.055
Cannabis	114 (23.1)	74 (27.8)	40 (17.6)	0.57	0.37, 0.89	0.056
Cocaine	171 (34.7)	97 (36.5)	74 (32.6)	0.80	0.54, 1.17	0.417
Ecstasy	23 (4.7)	13 (4.9)	10 (4.4)	1.00	0.69, 1.44	0.985
Positive urine screens in prior three months, percent; median (*Q*_1_, *Q*_3_)						
Amphetamines	0 (0, 0)	0 (0, 0)	0 (0, 0)	−0.56	−1.11, −0.01	0.141
Benzodiazepines	0 (0, 27.3)	0 (0, 20.0)	0 (0, 30.8)	−0.00	−0.27, 0.27	1.008
Cannabis	0 (0, 100.0)	0 (0, 100.0)	0 (0, 50.0)	−16.55	−26.90, −6.19	0.011
Cocaine	0 (0, 12.5)	0 (0, 17.7)	0 (0, 7.7)	−0.20	−0.52, 0.12	0.397
Opioids	0 (0, 25.0)	0 (0, 27.8)	0 (0, 20.0)	−0.21	−0.45, 0.06	0.273
Alcohol use disorder, M.I.N.I.; *n* (%)						
Alcohol dependence	26 (6.3)	14 (6.5)	11 (5.7)	0.81	0.34, 1.90	0.809
Alcohol abuse	13 (3.2)	7 (3.2)	6 (3.1)	0.84	0.27, 2.58	0.844
Smoking behavior, self-report						
Current smokers; *n* (%)	412 (84.1)	212 (80.0)	199 (88.8)	1.93	1.14, 3.27	0.053
Cigarettes smoked daily; mean (SD)	16.9 (10.4)	18.3 (11.7)	15.4 (8.6)	−2.81	−4.79, −0.84	0.024
Age of first smoking, years; mean (SD)	15.5 (5.4)	15.5 (5.5)	15.4 (5.3)	0.11	−0.91, 1.12	0.909
Sensitivity analysis						
Proportion of use in prior month, MAP; *n* (%)						
Alcohol	227 (46.0)	129 (48.5)	97 (42.9)	0.73	0.50, 1.05	0.213
Heroin	57 (11.5)	35 (13.2)	21 (9.3)	0.54	0.29, 1.00	0.142
Illicit methadone	26 (5.3)	11 (4.1)	14 (6.2)	1.43	0.60, 3.41	0.635
Illicit benzodiazepines	53 (10.7)	29 (10.9)	23 (10.2)	0.90	0.49, 1.66	0.879
Cocaine	89 (18.0)	42 (15.8)	46 (20.4)	1.53	0.91, 2.59	0.241
Crack	57 (11.5)	31 (11.7)	25 (11.1)	0.86	0.42, 1.77	0.839
Amphetamines	32 (6.5)	16 (6.0)	15 (6.6)	0.39	0.10, 1.50	0.330
Cannabis	241 (46.9)	143 (53.8)	88 (41.2)	0.49	0.34, 0.72	<0.001

All analyses have been adjusted for age, methadone dose, and duration of treatment using multivariable regression and for multiple testing error using false discovery rate; results for binary variables reported as OR and results for continuous variables reported as MD. Variables with non-normal distribution (positive drug urine screens) have been log transformed for analysis; differences are reported on the log scale. Data for alcohol use disorder measured by the MINI was only available for 409 participants

*Q*_*1*_ quartile 1, *Q*_*3*_ quartile 3, *M.I.N.I.* Mini International Neuropsychiatric Interview, *SD* standard deviation, *MAP* Maudsley Addiction Profile, *OR* odds ratio, *MD* mean difference, *CI* confidence interval

**Fig. 2:**
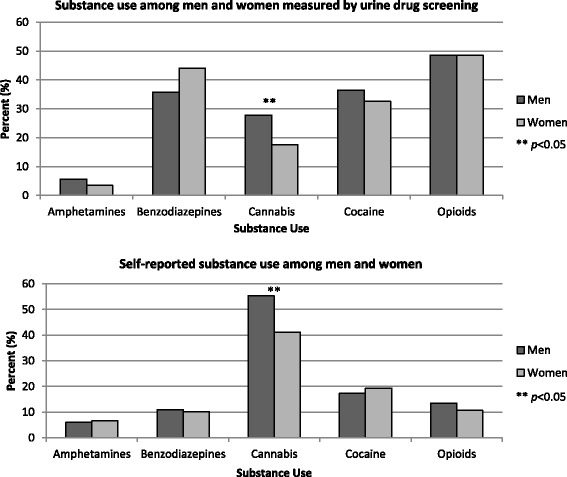
Comparison of substance use behavior among men and women measured by urine drug screening and self-report

Men and women were similar in their rates of opioid use measured through urine drug screening within the last 3 months (48.5 % for both). Cannabis use in the past 3 months was less likely among women compared to men (17.6 % vs. 27.8 %), and women had significantly fewer positive cannabis urine screens (MD = −16.55; 95 % CI = −26.90, −6.19; *p* = 0.011) (Table 2). These results were consistent when assessed using the MAP (Fig. 2). Women also reported more frequent use of benzodiazepines compared to men (44.1 % vs. 35.7 %; OR = 1.60; 95 % CI = 1.10, 2.33; *p* = 0.055).

Although alcohol use was reported more frequently among men within the past month (48.5 % vs. 42.9 %), no differences in alcohol-related disorders among men and women were observed when assessed by the M.I.N.I. (Table 2). In total, 84 % of the participants were current smokers, and women reported smoking significantly fewer cigarettes daily compared to men (15.4 vs. 18.3; MD = −2.81; 95 % CI = −4.79, −0.84; *p* = 0.024) (Table 2). No sex differences among other substance use were observed.

### Health status

Patterns of physical health symptoms demonstrate that over a third of the sample reported chronic pain (35.0 %) and a quarter of participants self-reported hepatitis C (24.7 %); 10.4 % of the sample reported both. In contrast, HIV rates were very low (0.8 %, *n* = 4) (Table 3). Scores for physical and psychological symptoms, measured by the MAP, were also low (15.8 and 13.3 out of 40, respectively). Apart from reporting unprotected sex (42.9 %), health risk behavior was not frequently reported.

**Table 3:** Health and social functioning among men and women

		Sex		Adjusted analyses, men vs. women		
Outcome	Total	Men	Women	OR/MD	95 % CI	Adjusted *p*
	*n* = 492	*n* = 266	*n* = 226			
Physical health symptoms						
MAP physical symptoms score; mean (SD)	15.8 (7.7)	14.5 (7.8)	17.4 (7.3)	3.18	1.83, 4.53	<0.001
HIV+ status; *n* (%)	4 (0.8)	4 (1.5)	0 (0)	--	--	--
HCV+ status; *n* (%)	122 (24.7)	73 (27.4)	49 (21.7)	0.88	0.57, 1.38	0.815
Presence of chronic pain; *n* (%)	173 (35.0)	94 (35.3)	78 (34.5)	1.31	0.87, 1.97	0.368
Mental health symptoms						
MAP psychological symptoms score; mean (SD)	13.3 (8.8)	12.0 (8.4)	14.7 (9.1)	2.77	1.20, 4.34	0.007
Family psychiatric history; *n* (%)	350 (70.9)	167 (62.8)	182 (80.5)	2.36	1.53, 3.62	<0.001
Health risk behavior in the prior month						
Injected drugs; *n* (%)	53 (10.8)	34 (12.8)	19 (8.4)	0.56	0.30, 1.02	0.146
Unprotected sex; *n* (%)	212 (42.9)	117 (44.0)	95 (42.0)	0.80	0.55, 1.18	0.426
Employment						
Currently employed; *n* (%)	175 (35.6)	114 (42.9)	61 (27.1)	0.46	0.31, 0.68	<0.001
Paid work in the past month, days; median (*Q*_1_, *Q*_3_)	0 (0, 16)	8 (0, 20)	0 (0, 4)	−0.04	−0.21, 0.13	0.825
Unemployed in the past month, days; median (*Q*_1_, *Q*_3_)	30 (0, 30)	30 (0, 30)	30 (0, 30)	0.02	−0.04, 0.07	0.828
Criminal activity						
Committed crime; *n* (%)	26 (5.3)	18 (6.8)	8 (3.5)	−0.04	−0.08, 0.00	0.148
Interpersonal relations						
Married/common-law; *n* (%)	156 (31.8)	85 (32.1)	70 (31.2)	0.94	0.63, 1.39	0.855
Have children; *n* (%)	309 (62.9)	144 (54.1)	164 (73.2)	2.88	1.90, 4.36	<0.001
Conflict with partner in the past month, percent; median (*Q*_1_, *Q*_3_)	0 (0, 7)	0 (0, 3)	0 (0, 10)	0.11	−0.29, 0.50	0.078
Conflict with family in the past month, percent; median (*Q*_1_, *Q*_3_)	0 (0, 7)	0 (0, 3)	0 (0, 13)	0.42	0.05, 0.80	0.800

All analyses have been adjusted for age, methadone dose, and duration of treatment using multivariable regression and for multiple testing error using false discovery rate; results for binary variables reported as OR and results for continuous variables reported as MD. Regression model estimates for HIV+ status were undeterminable

*MAP* Maudsley Addiction Profile, *SD* standard deviation, *HIV* human immunodeficiency virus, *HCV*: hepatitis C virus, *Q*_*1*_ quartile 1, *Q*_*3*_ quartile 3, *OR* odds ratio, *MD* mean difference, *CI* confidence interval

Women endorsed symptoms of physical and psychological illness more frequently than men, observed by significantly greater scores on the MAP health domains; 17.4 vs. 14.5 for physical health (MD = 3.18; 95 % CI = 1.83, 4.53; *p* < 0.001), and 14.7 vs. 12.0 for psychological health (MD = 2.77; 95 % CI = 1.20, 4.34; *p* = 0.007) (Table 3). Women were also significantly more likely to report a family psychiatric history compared to men (OR = 2.35; 95 % CI = 1.53, 3.62; *p* < 0.001). A greater proportion of men reported positive HIV status, but rates of hepatitis C (27.4 % in men vs. 21.7 % in women) and chronic pain (35.3 % for men vs. 34.5 % for women) were equally prevalent among men and women (Table 3).

### Social functioning

Among our participants, 35.6 % reported current employment; the median number of days worked in the past month was 8 for men and 0 for women (Table 3). Criminal activity within the past month was rare (5.3 %). Less than half of participants were married (31.8 %) and a majority reported having children (62.9 %).

Women were less likely to report current employment compared to men (27.1 % vs. 42.9 %; OR = 0.46; 95 % CI = 0.31, 0.68; *p* < 0.001) but were more likely to report having children to care for (73.2 % vs. 54.1 %; OR = 2.88; 95 % CI = 1.90, 4.36; *p* < 0.001) (Table 3).

## Discussion

The results of this study confirm that trends in illicit opioid use in Canada are undergoing dynamic changes, giving rise to a new sociodemographic profile of opioid users. Compared to past literature, the mean age of current opioid-dependent patients enrolled in MMT has increased from 25 to 38 years [21, 32, 33], starting regular use of opioids later (25 years of age now compared to 21 years in the 1990s) and entering treatment at a later age than before (currently 32 years compared to 27 years of age) [17, 21, 33–35]. There has been a 30 % increase in the proportion of patients who began using opioids after receiving a prescription from a doctor (20 % in the 1960s to 50 %) [17], usually for the management of chronic pain, which was present in a third of patients. We also observed an approximate 60 % decrease in injection drug use [35, 36] and 50 % reduction in rates of HIV [37]. We have witnessed a gradual deviation from alcohol use to cannabis [34, 35] and greater rates of benzodiazepine use [38]. Criminal activity has also declined significantly compared to earlier studies (34 % to 5 % among current opioid users) [35, 38].

Women, who are close to half of the opioid user population, experience a higher burden of disease related to opioid use disorders, with respect to physical and psychological disorders and related symptoms. Women are more likely to have initiated their substance dependence through prescription opioids, presumably because of their higher rates of chronic pain [13]. Indeed, women are known to experience heightened pain perception and sensitivity and to have lower levels of opioid analgesia compared to men [39]. This disparity in opioid prescribing may also be attributed to the utilization of healthcare services, as women tend to seek medical care for pain-related conditions more often than men [40]. Heroin use is also decreasing as a result of this dependence on prescription opioids among women, and therefore we are witnessing lower rates of HIV that normally would have been caused by unsafe heroin injection practices.

Cannabis is now the most prevalent drug of abuse in North America, even though it remains illegal across Canada and most of the USA [9]. Given the considerable rate of chronic pain among participants, it is possible that cannabis is being used as an adjunctive therapy to manage pain. Women are less likely to use cannabis than men, consistent with earlier investigations [41, 42], which may be attributed to the social stigma associated with substance use among women. Alternatively, women may be deterred from using cannabis because of the potential legal implications of this behavior.

Although both alcohol and cocaine use continue to be problematic among opioid users in MMT, the disparity that has been seen in the past, with men more likely to abuse alcohol [33, 34] and women more likely to abuse cocaine [41–43], is less apparent. Alcohol use has become a concern for women as well, perhaps because of changes in social roles and attitudes regarding its use [44, 45]. Also, it is expected that cocaine use is decreasing as it caters primarily to a younger inner-city group of users, which is characteristic of the former opioid user population [46].

In comparing self-reported substance use measured by the MAP to urine toxicology screening in our sensitivity analysis, we observed considerable under-reporting of benzodiazepine, cocaine, and opioid use. This finding is likely a result of social desirability bias, or in the case of benzodiazepine use, it may also come from a prescription for an anxiety-related disorder, although we do not have the data to confirm this. Overall, objective measures, such as urine screening, are more reliable than self-report in identifying drug use in men and women alike and should be used regularly across all methadone programs and in future research studies, if possible.

Women experience a heightened vulnerability to the adverse medical and social consequences of opioid dependence [35, 47] as a result of biological sex characteristics and socially defined gender roles. Although sex and gender differences in MMT have been previously investigated, the literature is limited by the scarcity of studies, poor methodological quality, and small samples. A recent systematic review and meta-analysis found 20 studies, many of which were completed over a decade ago, specifically evaluating methadone treatment outcomes among men and women [48]. Men were more likely to be employed and to report a history of legal involvement and alcohol-related problems, and women were more likely to have used illicit amphetamines throughout the course of treatment [48]. Sex differences in physical health [35], comorbid psychiatric conditions [34, 49, 50], and substance use behavior [33, 34, 41, 42, 51] have also been documented, however findings appear to be conflicting and generally based on subjective self-reported measures.

### Implications and future directions

Based on the documented changes in the illicit use of opioids, prevention strategies and modifications to available opioid addiction treatment programs are needed. Current treatments were initially developed using research from the 1990s targeting heroin users [52], and thus their applicability to the growing population of prescription opioid users is questioned. Guidelines for the treatment of opioid addiction with methadone [53] require a thorough re-evaluation to incorporate this transition and the implementation of new intervention strategies that address the evolving trends in substance use, health, and social functioning is strongly encouraged.

Women also experience a greater burden of disease from opioid dependence with respect to medical problems, health outcomes, and social impairment, elucidating the need for interventions that address these core areas of functioning for women [54]. Currently available best practice guidelines for methadone maintenance treatment in Canada outline barriers to treatment and highlight areas for improvement, however these recommendations rely largely on a small and weak body of evidence comprised of outdated literature reviews. Similarly, the US federal guidelines for opioid treatment programs and medication-assisted treatment developed by Department of Health and Human Services, Substance Abuse and Mental Health Services Administration (SAMHSA) [55] acknowledge that women require specialized treatment services, however they are not sufficiently comprehensive as the focus is primarily on pregnancy, physical or sexual abuse, and complex medical problems in women. Furthermore, guidelines for pharmacologically assisted treatment of opioid dependence set forth by the World Health Organization in 2009 [56] acknowledge areas where women experience particular difficulty and they emphasize the need for gender-sensitive treatment services but admit that data on such programs are sparingly available.

First, it is necessary to implement appropriate prevention strategies in general but especially for women. As our results have shown, women are more likely to be exposed to opioids mainly through prescriptions for pain and other medical conditions. This information can be used to inform both patients and physicians and in the assessment of individual benefit or risk of opioid-related harms. Alternative treatment and therapeutic options should be considered in the management of pain conditions that require the use of opioid analgesics.

Behavioral therapy and social services can supplement current pharmacological treatment programs in order to develop an integrated patient-centered model of care. Emphasizing the need for fundamental services, such as vocational counseling, childcare and parenting assistance, medical assistance, relationship or domestic violence counseling, and smoking cessation among women is likely to significantly improve the treatment and management of opioid use disorder [57]. Similar strategies should be implemented for men in treatment, who experience distinct sex- and gender-specific characteristics of addiction (i.e., HIV, cannabis and amphetamine use). This field of research would benefit from future studies that evaluate the efficacy of these programs compared to standard care and assess patient-important outcomes that can be incorporated into a personalized treatment approach.

### Strengths and limitations

This study is limited by its cross-sectional design, whereby sociobehavioral determinants of opioid use disorder were assessed at a single time-point that captured a period of 30 days (or 3 months in the case of urine screening). A longer time frame would be more appropriate considering the chronicity of the illness and long treatment duration [58]. In addition, some of the trends we observed in this study may be attributed to general population differences rather than the specific context of opioid users in methadone treatment. Nevertheless, such factors are still an important consideration for treatment among men and women.

Despite these limitations, our study had numerous strengths. We offer a comprehensive update of factors characterizing a large sample of opioid users receiving methadone treatment within the Canadian context. Our study also provides a descriptive profile of sex differences in methadone treatment, clarifying previous gaps in the literature. We used an objective measure of urine toxicology and performed a sensitivity analysis using self-reported substance use in order to strengthen credibility in our findings. Based on our results, the response rate for MMT in this sample is generally comparable to other studies in the literature (30–80 % of opioid urine screens generally test negative [53–55]), confirming the representativeness of this sample. Finally, our data were derived from a multisite study, whereby standardized treatment procedures are implemented across all 13 clinic sites, yielding a large representative and geographically diverse sample.

## Conclusions

The results of this study have revealed new patterns in substance use, health, and social factors among men and women currently receiving MMT for opioid use disorder in Ontario, Canada. We have uncovered clinically relevant sex differences that can be used to advance our understanding of addiction and promote strategies for effective treatment and management of opioid use disorder among men and women.

## Abbreviations

CATC: Canadian Addiction Treatment Centres
CI: confidence interval
GENOA: Genetics of Opioid Addiction
HIREB: Hamilton Integrated Research Ethics Board
M.I.N.I.: Mini International Neuropsychiatric Interview
MAP: Maudsley Addiction Profile
MD: mean difference
MMT: methadone maintenance treatment
OATC: Ontario Addiction Treatment Centres
OR: odds ratio
PGP: Population Genomics Program
SAMHSA: Substance Abuse and Mental Health Services Administration
SD: standard deviation
STROBE: Strengthening the Reporting of Observational Studies in Epidemiology
